# Neurons of the median preoptic nucleus contribute to chronic angiotensin II‐salt induced hypertension in the rat

**DOI:** 10.14814/phy2.15551

**Published:** 2022-12-23

**Authors:** John P. Collister, Trasida Ployngam, Pilar A. Ariza‐Guzman, John W. Osborn

**Affiliations:** ^1^ Department of Veterinary and Biomedical Sciences, College of Veterinary Medicine University of Minnesota St. Paul Minnesota USA; ^2^ Department of Integrative Biology and Physiology University of Minnesota Minneapolis Minnesota USA; ^3^ Department of Surgery University of Minnesota Minneapolis Minnesota USA

## Abstract

Experiments were designed to test the hypothesis that median preoptic (MnPO) neurons are necessary for the full hypertensive response to chronic angiotensin II (AngII) in rats consuming a high salt diet. The MnPO is implicated in many of the physiologic actions of AngII, primarily acting as a downstream nucleus to AngII binding at circumventricular organs such as the organum vasculosum of the lamina terminalis (OVLT). We have previously shown a prominent effect of lesion of the OVLT on the chronic hypertensive effects of AngII in rats consuming high salt. Additionally, we have shown that lesion of the MnPO attenuated the hypertensive response to chronic intravenous infusion of AngII in rats. However, whether MnPO neurons or fibers of passage contribute to this response is not clear. Male Sprague Dawley rats were randomly assigned to either sham (SHAM; *n* = 8) or ibotenic acid lesion of the MnPO (MnPOx; *n* = 6). In the MnPOx group, 200 nl of ibotenic acid in phosphate buffer saline (5 μg/μl) was injected into each of 3 predetermined coordinates targeted at the entire MnPO. After a week of recovery, rats were instrumented with radiotelemetric pressure transducers, provided 2.0% NaCl diet and distilled water ad libitum and given another week to recover. After 3 days of baseline measurements, osmotic minipumps were implanted subcutaneously in all rats for administration of AngII at a rate of 150 ng/kg/min. Blood pressure measurements were made for 14 days after minipump implantation. By day 7 of AngII treatment, blood pressure responses appeared to plateau in both groups while the hypertensive response was markedly attenuated in MnPOx rats (MnPOx, 122 ± 6 mmHg; SHAM, 143 ± 8 mmHg). These results support the hypothesis that neurons of the MnPO are involved in the central pathway mediating the chronic hypertensive effects of AngII in rats consuming a high salt diet.

## Graphical abstract

Previously we have shown electrolytic lesion of the MnPO prevents chronic angiotensin II hypertension in the rat. Currently, by utilizing chemical lesion of the MnPO sparing fibers of passage, we demonstrate specifically that neurons of the MnPO are necessary for the full hypertensive response to angiotensin II in rats consuming a high salt diet.

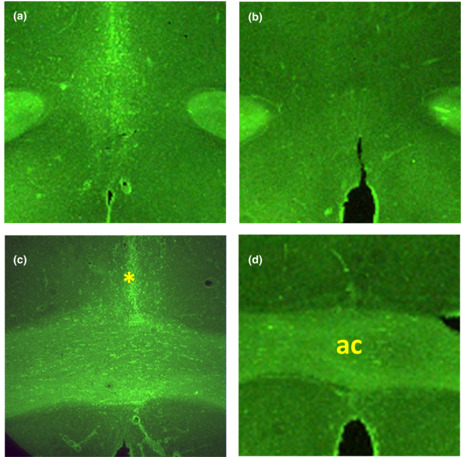

## 1 INTRODUCTION

It is well established that chronic angiotensin II (AngII) treatment causes a sustained elevation of arterial pressure in the rat (Collister & Hendel, 2005; Hendel & Collister, 2005; Vieira, Nahey, & Collister, 2010), and much evidence supports the notion that there is a large neurogenic and sympathetically mediated component to this hypertensive model (Brody et al., 1978; Osborn & Fink, 2010; Osborn, Fink, & Kuroki, 2011; Osborn, Fink, Sved, Toney, & Raizada, 2007). Furthermore, the rise in blood pressure attributable to sympathetic nervous system activation is thought to be exacerbated by increased dietary salt (Collister, Olson, Nahey, Vieira, & Osborn, 2013; Osborn et al., 2007, 2011; Osborn, Hendel, Collister, & Fink, 2012). Therefore, it is important to distinguish between “AngII hypertension” and “AngII‐salt induced hypertension” as the central nervous system components and pathways activated and responsible for sympathoexcitation resulting from a rise in circulating AngII may not be identical to those that are responsive to a combination of increased AngII and sodium. While there is agreement that ultimately an increase in sympathetic nervous system activity accompanies chronic AngII treatment in both models and leads to hypertension, the central signaling sites and pathways involved in this response are not fully understood.

Historically, the anterior and ventral portions of the tissue relative to the third ventricle (AV3V) has been implicated in a number of studies as being critical for sodium and water homeostasis (Buggy & Brody, 1977; Sladek & Johnson, 1983). This relatively large AV3V is comprised of the organum vasculosum of the lamina terminalis (OVLT), median preoptic nucleus (MnPO) and efferent fibers of the subfornical organ (SFO). Furthermore, this entire area has been shown to be involved not only in sodium and water homeostasis, but blood pressure regulation as well, as its ablation prevents many forms of experimental hypertension (Brody et al., 1978; Buggy, Fink, Johnson, & Brody, 1977).

Two components of the aforementioned AV3V, the SFO and OVLT, are unique structures known as circumventricular organs (CVO), and as such lack the normal blood brain barrier (Johnson & Loewy, 1990) and have been shown to be responsive to circulating AngII (Mangiapane & Simpson, 1980a, 1980b; McKinley et al., 1992; McKinley, Allen, Burns, Colvill, & Oldfield, 1998). Furthermore, both of these CVO project to the MnPO (McKinley et al., 1992; Miselis, 1981), a hypothalamic relay station with projections to the paraventricular nucleus (PVN) (Xu & Herbert, 1995), which subsequently projects to sympathetic premotor neurons of the rostral ventral lateral medulla and preganglionic sympathetic cell bodies of the spinal cord (Bains & Ferguson, 1995; Stocker & Toney, 2005). Our lab has investigated each of these 3 components of the AV3V region and their individual roles in mediating the effects of chronic “AngII hypertension” and have reported that discrete lesions of each of these areas (SFO, OVLT and MnPO) attenuated or nearly abolished “AngII hypertension” (Collister & Hendel, 2005; Hendel & Collister, 2005; Ployngam & Collister, 2007; Vieira et al., 2010).

Additionally, with regard to “AngII‐salt induced hypertension,” and the synergistic effects of AngII and a high salt diet, we initially investigated a role of the SFO in this model as others had reported a dominant role of the SFO in the mouse model of “AngII‐salt induced hypertension” (Young et al., 2012; Zimmerman, Lazartigues, Sharma, & Davisson, 2004). Surprisingly contrary to our hypothesis, we reported that SFO lesion had minor effects on the overall magnitude of the “AngII‐salt induced hypertension” (Osborn et al., 2012), and the attenuated rise in blood pressure was similar to that observed in our earlier study using normal salt in the “AngII hypertension” model (Hendel & Collister, 2005).

Based on further reports that the OVLT is a prominent central osmoreceptor, as it has been shown to be involved in mediating sympathetic nervous system activity in response to acute increases in plasma osmolality (Toney, Chen, Cato, & Stocker, 2003; Toney & Stocker, 2010), we tested the hypothesis that the OVLT is necessary for the full rise in arterial pressure in the “AngII‐salt induced hypertension” model. Unlike our reported results with SFO lesions in this model, OVLT lesion had a pronounced effect in decreasing the overall magnitude of the hypertensive response to AngII and increased dietary salt (Collister et al., 2013), suggesting a prominent role of this CVO in mediating the combined synergistic effects of these 2 central signals.

As mentioned above, the OVLT has prominent neural projections to and passing through the MnPO (McKinley et al., 1992), which ultimately project to sympathetic regulating neurons of the PVN. Furthermore, we have previously reported a role of MnPO neurons in “AngII hypertension (Ployngam & Collister, 2008). Therefore, the present study was designed to test the hypothesis that neuronal cell bodies of the MnPO are necessary and critical for the full hypertensive response to AngII and increased dietary salt (i.e. “AngII‐salt induced hypertension”). To that end, cytotoxic lesions specific to cell bodies were induced in the MnPO using ibotenic acid injections, and the hypertensive responses to 14 days of AngII – high salt treatment were measured and contrasted with those of vehicle lesioned rats.

## 2 MATERIALS AND METHODS

Adult male Sprague Dawley rats (Charles River Laboratory, Wilmington, MA, USA) weighing 300‐350 g were used in all procedures. All procedures were approved by University of Minnesota Institutional Animal Care and Use Committee in accordance with the National Institutes of Health guidelines.

### 2.1 Surgical procedures

Rats were randomly assigned to either MnPO‐lesioned (MnPOx; *n* = 6) or sham‐lesioned (SHAM; *n* = 8) group. Surgical anaesthesia was achieved with an injection of pentobarbital sodium (50 mg/kg, IP). All rats were given a prophylactic antibiotic injection of 4 mg intramuscular tobramycin. Anaesthetized rats were then placed in a Kopf stereotaxic apparatus (Kopf Instruments, Tujunga, CA, USA). Ibotenic acid lesions were aimed at the entire MnPO and performed as previously described (Ployngam & Collister, 2008; Ployngam, Katz, & Collister, 2010). Briefly, a dorsal midline incision was made through the skin of the skull, bregma and lambda landmarks were levelled in the same horizontal plane, and a 3 mm hole centred over bregma was drilled through the skull. The three‐paired anterior‐posterior and dorsal‐ventral coordinates (mm) used to target the MnPO relative to bregma and the surface of the sagittal sinus were as follows: (−0.25, −7.4), (−0.4, −6.1), and (−0.35, −7.2). An injection cannula (0.15 mm inner diameter; 0.30 mm outer diameter; Plastic Ones, Roanoke, VG, USA) was lowered along the midline to each coordinate and 200 nl ibotenic acid (Sigma Aldrich, St Louis, MO, USA) in 1 M phosphate‐buffered saline (PBS; 5 μg/μL) was injected over a period of 10 minutes. The injection cannula was left in place for an additional 10 min before being withdrawn. Vehicle sham injections were performed identically to lesions, except that the cannula was lowered 2 mm less and no acid was infused.

Seven days after ibotenic acid lesion or sham injection rats underwent implantation of radiotelemetry blood pressure transducers (model TA11PAC40, Data Sciences International, St Paul, MN) for 24‐h sampling of mean arterial pressure (MAP) and heart rate (HR). This technique has been described previously (Ployngam & Collister, 2007). The telemetry unit consists of a fluid‐filled catheter attached to the body of the transmitter/transducer. An abdominal incision was performed to insert the body of the transducer into the abdomen. The telemetry catheter was passed through the abdominal wall, inserted into the femoral artery, and advanced proximally such that the tip was situated within the abdominal aorta distal to the renal arteries. Immediately after surgery, rats received a subcutaneous injection of 0.075 mg of butorphanol tartrate for analgesic purposes.

All rats were housed in individual metabolic cages (Nalgene; Nalge Nunc International, Rochester, NY, USA) in a housing facility that was maintained at a temperature of approximately 23°C with a 12 h:12 h light–dark cycle with lights on at 7:00 AM. Rats had free access to 2.0% NaCl diet (Research Diets, New Brunswick, NJ, USA) and distilled water. After instrumentation, rats were allowed to recover for at least 1 week before the experimental protocol was begun.

### 2.2 Experimental protocol

MAP and HR were sampled using Dataquest V software (Data Sciences International, St. Paul, MN) every 4 min for 10 s throughout the protocol. The experimental protocol ran for 17 days and consisted of 3 days of baseline measurements followed by 14 days of continuous AngII (Sigma Chemical Corp., St. Louis, MO) treatment. AngII was administered subcutaneously at a rate of 150 ng/kg/min by osmotic minipump (Model 2ML2, Durect Corp, CA). The minipump implantation was performed under brief isoflurane anesthesia immediately following measurements on day 3 of the protocol. This dose and route of AngII administration was chosen for comparison with findings of our previous studies (Osborn et al., 2011, 2012; Osborn & Fink, 2010), and others have reported that this protocol results in a small increase of plasma AngII that is well within the physiological range (Huang, Ahmadi, Ahmad, White, & Leenen, 2010).

### 2.3 Lesion verification

**FIGURE 1:**
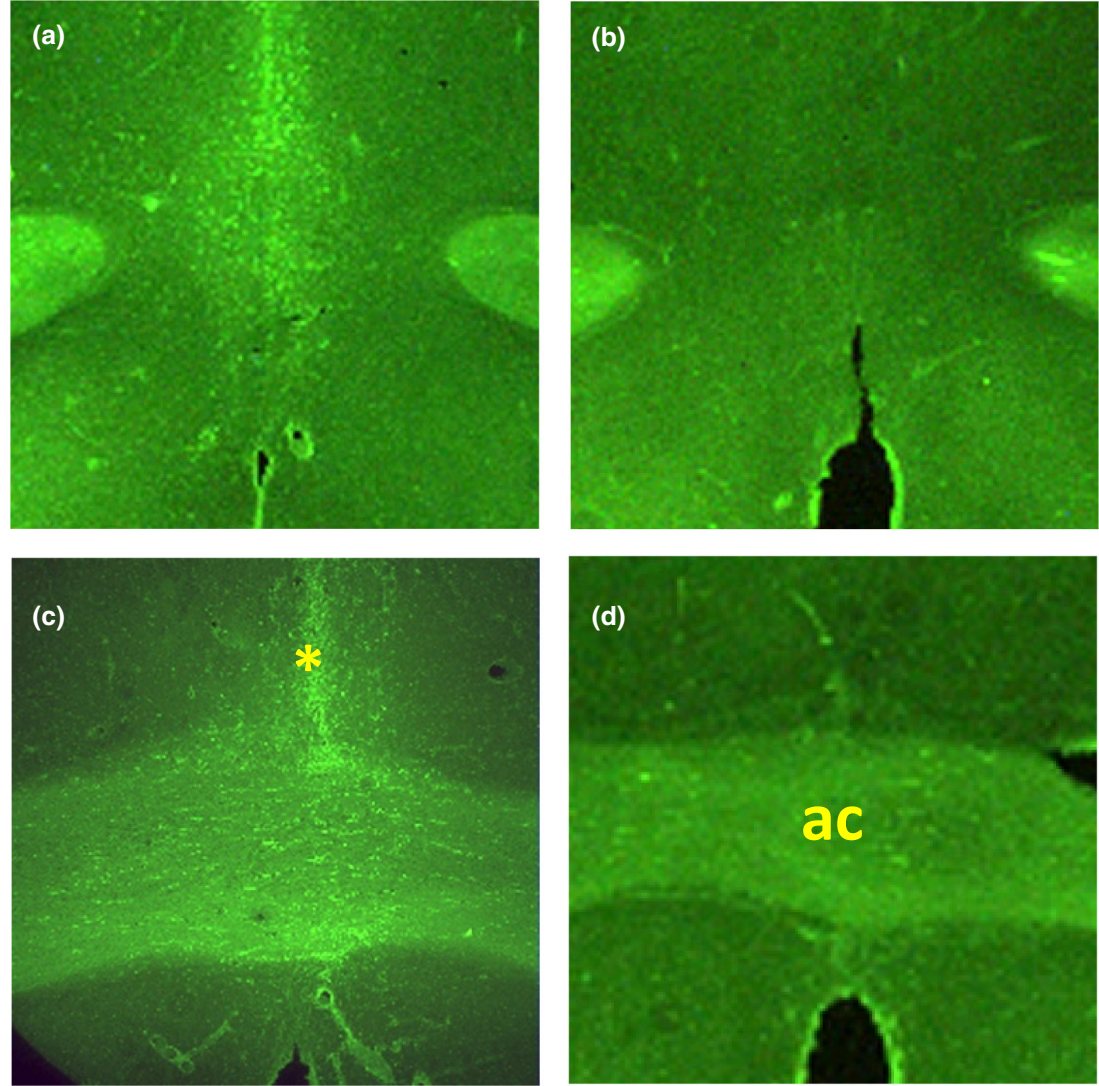
Photomicrographs of 40 μm coronal sections through the region of the median preoptic nucleus (MnPO). Sections of anterior part of the MnPO from an ibotenic acid lesion rat (MnPOx) (A) and a sham lesion rat (SHAM) (B). Sections of dorsal (*) parts of the MnPO from MnPOx (C) and SHAM (D). Note degenerating neurons and synapsing fibers are stained with fluorescent stain Fluoro‐jade‐C in the entire MnPO in MnPOx compared to an absence of stained area in the SHAM. ac, Anterior commissure.

At the end of the protocol, rats were anesthesized and perfused transcardially with 4% paraformaldehyde in phosphate buffered saline. The brains were post‐fixed with 4% paraformaldehyde overnight and in 30% sucrose for another 48 h for cryoprotection. Coronal sections of the area comprising the entire MnPO were cut on a sliding, freezing stage microtome (Lipshaw Mfg, Detroit, MI) at a thickness of 40 μm. Sections were stained with Fluoro‐Jade‐C (Chemicon International Inc, Temecula, CA), a fluorescent, anionic stain that labels degenerating neurons. Fluoro‐Jade staining was performed as described previously (McLin & Steward, 2006; Schmued, 2000). Briefly, sections were mounted on gelatin‐coated slides, dried overnight, and the slides were then immersed in 100% ethanol for 3 min, followed by 70% ethanol for 1 min, and finally dH_2_O for 1 min. Sections were then treated with 0.06% potassium permanganate for 15 min, while shaking gently. After rinsing with dH_2_O, sections were immersed in 0.001% Fluoro‐jade‐C in 0.1% acetic acid for 30 min in the dark and then rinsed with dH_2_O. The sections were dried overnight, immersed for 3 min in xylene substitute (Fisher Scientific, Fair Lawn, NJ), and coverslipped with the nonaqueous fluorescent mounting media DPX (Sigma‐Aldrich, St. Louis, MO). Fluoro‐Jade‐stained sections were visualized with a fluorescence microscope using a FITC‐filter (Nikon Instruments Inc, New York, NY). Only MnPOx rats that had at least 90% of the MnPO area positively stained with Fluoro‐jade‐C, with no or few degenerate neurons observed in adjacent areas were included in the final data analyses (Figure 1).

### 2.4 Statistical analyses

All statistical procedures were performed using NCSS software (NCSS, Kaysville, UT). Two‐way ANOVA with repeated measures was carried out to compare each cardiovascular parameters between MnPOx and SHAM groups. Post‐hoc multiple comparisons using a Tukey–Kramer test was further conducted to identify days on which the two groups differed. Baseline values were derived from averages over the three control days, and the differences of baselines between the two groups were determined using a Student's *t* test. A *p*‐value of 0.05 was set as the level of statistical significance for all statistical analyses. All values were presented as mean ± SE.

## 3 RESULTS

Fluoro‐Jade‐C staining confirmed 6 rats with complete lesion of the MnPO (MnPOx) and 8 rats with intact MnPO from the SHAM group which were included in the analyses.

### 3.1 Cardiovascular response to AngII and high dietary salt

**FIGURE 2:**
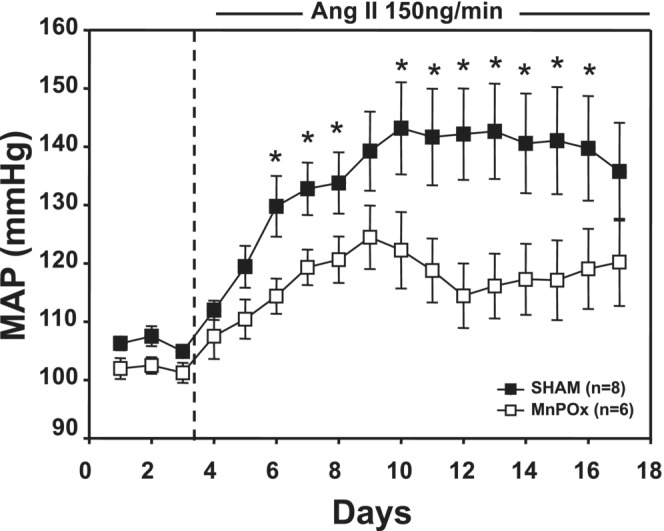
Average 24 h mean arterial pressure (MAP) during 3‐day control and 14‐day AngII treatment period in ibotenic acid lesion rats (MnPOx) and sham lesion rats (SHAM). *p < 0.05 between groups.

Figure 2 shows the MAP responses to AngII in MnPOx and SHAM lesion rats throughout the experimental protocol. No statistical difference in 3‐day baseline MAP was found between the two groups. The baseline 3‐day average MAP in MnPOx rats was 102 ± 2 and 106 ± 2 mmHg in SHAM rats. By day 2 of AngII treatment, MAP in both MnPOx and SHAM lesion rats were significantly increased from baseline control (MnPOx, 110 ± 3 mmHg; SHAM, 119 ± 4 mmHg). MAP continued to rise in both groups and yet the increase in arterial pressure was attenuated in MnPOx rats by day 3 of AngII treatment. By day 7 of AngII treatment blood pressure responses appeared to plateau in both groups while the hypertensive response was still markedly attenuated in MnPOx rats (MnPOx, 122 ± 6 mmHg; SHAM, 143 ± 8 mmHg). This trend continued throughout the rest of the treatment protocol.

HR was similar between MnPOx and SHAM groups throughout the experimental protocol. The 3‐day average baseline HR in MnPOx rats and SHAM lesion rats was 419 ± 7 and 413 ± 8 beats/min, respectively. HR was significantly decreased from baseline by day 4 of AngII infusion in MnPOx (400 ± 8 beats/min) and by day 3 in SHAM (390 ± 15 beats/min) rats. This modestly lower HR remained throughout the remainder of the protocol in both groups such that by day 14 of AngII treatment HR was 396 ± 11 beats/min in MnPOx rats and 391 ± 4 beats/min in SHAM rats.

## 4 DISCUSSION

We have previously demonstrated a role for the SFO, OVLT and MnPO in the chronic hypertensive effects of “AngII hypertension” as lesion of each individual central structure attenuated the hypertensive effect of AngII during normal salt intake (Collister & Hendel, 2005; Hendel & Collister, 2005; Ployngam & Collister, 2007; Vieira et al., 2010). Furthermore, we have demonstrated a large effect of OVLT lesion on the hypertensive effects of “AngII‐salt induced hypertension” as lesion of the OVLT markedly attenuated the chronic effects of AngII during high salt (Collister et al., 2013), while lesion of the SFO had minimal effects (Osborn et al., 2012).

Since the MnPO is a major relay station for OVLT neurons, containing both synapsing fibers to MnPO neurons as well as fibers of passage en route to the PVN (McKinley et al., 1992), we sought to determine the role of neuronal cell bodies of the MnPO in the chronic hypertensive effects of “AngII‐salt induced hypertension.” Our results demonstrated a marked attenuation of approximately 20 mmHg to the hypertensive effects of AngII during a high salt diet. Therefore, these findings suggest that MnPO neurons are a critical component of the central nervous system signaling pathway mediating the hypertensive effects of chronic AngII administration during increased dietary salt. These results were strikingly similar with our findings in rats with lesions of the OVLT during “AngII‐salt induced hypertension” (Collister et al., 2013). In that previous study, we reported OVLT lesioned rats had a similarly attenuated hypertensive response of approximately 18 mmHg during chronic AngII administration and increased dietary salt. Therefore, these findings together with the present results suggest that the OVLT itself along with efferent projections to the cell bodies of the MnPO are responsible for a large portion of the of the hypertensive response during “AngII‐salt induced hypertension”.

While lesion of the MnPO in this study reduced the chronic pressor response to AngII and high salt by nearly 50%, there still remained a prominent pathway causing a hypertensive response. While there may be fibers of passage from activated SFO neurons in the MnPO capable of mediating this response (Miselis, 1981), we do not believe these to be responsible as our previous studies in rats with lesions of the SFO showed minimal attenuation of hypertension during “AngII‐salt induced hypertension” (Osborn et al., 2012). Furthermore, even though the present findings were similar to the attenuated hypertensive effects we reported in OVLT lesioned rats during “AngII‐salt induced hypertension” (Collister et al., 2013), OVLT fibers of passage through the MnPO remained intact in the current study and could therefore compensate for the remaining hypertensive effects of the synergistic AngII‐high salt signal. This is supported by the fact that we have previously reported a greater effect of electrolytic lesion of the MnPO compared to ibotenic acid lesion of the MnPO during chronic “AngII hypertension” (Ployngam & Collister, 2007, 2008). This notion is also supported by the fact that the OVLT has been shown to be important for mediating the enhanced pressor and sympathetic nerve activity responses to the combination of increased salt and AngII via an OVLT‐paraventricular nucleus‐RVLM pathway (Toney & Stocker, 2010), as increased arterial pressure and sympathetic nerve activity in response to activation of sympathetic premotor neurons in the RVLM in rats consuming a high salt diet is prevented by lesion of the OVLT (Adams, Bardgett, & Stocker, 2009). Lastly, we have previously shown that MnPO lesioned rats consuming a high salt diet indeed have elevated plasma sodium and osmolality compared to sham controls (Ployngam, Katz, & Collister, 2012). Taken together, it is plausible that the remaining elevated pressure effects of “AngII‐salt hypertension” despite MnPO lesion in the present study are mediated through activated sodium sensing neurons of the OVLT which present fibers of passage through the MnPO to PVN and RVLM sympathetic control centers to maintain the remaining elevation in blood pressure.

In summary, a large body of evidence has established an important role for the MnPO in a variety of physiological central actions of AngII. Neuronal circuitry passing through or directly synapsing on MnPO neurons are necessary for drinking, vasopressin and acute increases in blood pressure in response to AngII (Cunningham et al., 1991; Cunningham, Beltz, Johnson, & Johnson, 1992; Gardiner & Stricker, 1985; Gutman, Jones, & Ciriello, 1989; Jones, 1988; Lind & Johnson, 1982; Lind, Ohman, Lansing, & Johnson, 1983; Mangiapane, Thrasher, Keil, Simpson, & Ganong, 1983; O'Neill & Brody, 1987). Additionally, we have previously shown that ibotenic acid lesion of the MnPO neurons attenuated increased pressure responses during “AngII hypertension”(Ployngam & Collister, 2008) demonstrating a role for neurons of the MnPO in that response. However, it was not clear whether neural cell bodies of the MnPO or fibers of passage were responsible for the observed blunted pressure response to “AngII‐salt induced hypertension”. In the current study, we have added to our previous findings of the specific and important role of MnPO neurons in the chronic hypertensive effect of AngII during high salt. In conclusion, the present study with our previous findings (Collister et al., 2013) provide strong evidence that necessitate the central roles of the OVLT and MnPO neurons in the central neural circuitry of “AngII‐salt induced hypertension”.

## FUNDING INFORMATION

This study was supported by National Heart Lung and Blood Institute grant RO1 HL‐072180.

## 5 ETHICS STATEMENT

Adult male Sprague Dawley rats (Charles River Laboratory, Wilmington, MA, USA) weighing 300‐350 g were used in all procedures. All procedures were approved by University of Minnesota Institutional Animal Care and Use Committee in accordance with the National Institutes of Health guidelines.
